# Heparin (GAG-hed) inhibits LCR activity of Human Papillomavirus type 18 by decreasing AP1 binding

**DOI:** 10.1186/1471-2407-6-218

**Published:** 2006-08-31

**Authors:** Rita Villanueva, Néstor Morales-Peza, Irma Castelán-Sánchez, Enrique García-Villa, Rocio Tapia, Ángel Cid-Arregui, Alejandro García-Carrancá, Esther López-Bayghen, Patricio Gariglio

**Affiliations:** 1 Departamento de Genética y Biología Molecular, Centro de Investigación y de Estudios Avanzados, Apartado Postal 14-740, México D.F. 07000, México; 2 Unidad de Investigación Biomedica en Cáncer, UNAM- Instituto Nacional de Cancerología, Av. San Fernando 22, México D.F. 14000, México; 3 Tumor Gene Therapy German Cancer Research Center (DKFZ) Im Neuenheimer Feld 280 69120-Heidelberg, Germany

## Abstract

**Background:**

High risk HPVs are causative agents of anogenital cancers. Viral E6 and E7 genes are continuously expressed and are largely responsible for the oncogenic activity of these viruses. Transcription of the E6 and E7 genes is controlled by the viral Long Control Region (LCR), plus several cellular transcription factors including AP1 and the viral protein E2. Within the LCR, the binding and activity of the transcription factor AP1 represents a key regulatory event in maintaining E6/E7 gene expression and uncontrolled cell proliferation. Glycosaminoglycans (GAGs), such as heparin, can inhibit tumour growth; they have also shown antiviral effects and inhibition of AP1 transcriptional activity. The purpose of this study was to test the heparinoid GAG-hed, as a possible antiviral and antitumoral agent in an HPV18 positive HeLa cell line.

**Methods:**

Using *in vivo *and *in vitro *approaches we tested GAG-hed effects on HeLa tumour cell growth, cell proliferation and on the expression of HPV18 E6/E7 oncogenes. GAG-hed effects on AP1 binding to HPV18-LCR-DNA were tested by EMSA.

**Results:**

We were able to record the antitumoral effect of GAG-hed *in vivo *by using as a model tumours induced by injection of HeLa cells into athymic female mice. The antiviral effect of GAG-hed resulted in the inhibition of LCR activity and, consequently, the inhibition of E6 and E7 transcription. A specific diminishing of cell proliferation rates was observed in HeLa but not in HPV-free colorectal adenocarcinoma cells. Treated HeLa cells did not undergo apoptosis but the percentage of cells in G_2_/M phase of the cell cycle was increased. We also detected that GAG-hed prevents the binding of the transcription factor AP1 to the LCR.

**Conclusion:**

Direct interaction of GAG-hed with the components of the AP1 complex and subsequent interference with its ability to correctly bind specific sites within the viral LCR may contribute to the inhibition of E6/E7 transcription and cell proliferation. Our data suggest that GAG-hed could have antitumoral and antiviral activity mainly by inhibiting AP1 binding to the HPV18-LCR.

## Background

Cervical cancer represents the second most frequent malignant tumour found in women worldwide, with an estimated frequency of approximately 440,000 new cases per year, corresponding to about 5.8% of global cancer incidence [1]. In countries like Mexico, cervical carcinoma stands as the leading cause of death among the female population, with 14 deaths per 100,000 women (15 years old or more), representing 34% of all new female cancer cases reported [2,3]. Human papillomaviruses (HPVs), especially the high risk types 16 and 18, have been identified as causative agents of at least 90% of cervical cancer cases and are also linked to more than 50% of other anogenital cancers [4]. The HPV genome consists of around 8000 base pairs (bp) of closed-circular double-stranded DNA containing up to nine genes, functionally divided into three regions: a long control region (LCR) covering about 10% of the genome, and early (E) and late (L) regions [4]. The regulation of viral gene expression is complex and is controlled by multiple cellular and viral transcription factors. Most of the regulation occurs within the LCR, which varies substantially in nucleotide composition between individual HPV types. Within the LCR, cis-active elements regulate transcription of the E6/E7 genes, which represent the transforming genes for immortalization and for maintenance of the malignant phenotype in HPV-positive cervical cancer cells [5-7].

A number of cellular transcription factors, such as NF1, AP1, KRF1, Oct1, SP1, YY1, and the glucocorticoid receptor, have been shown to bind and regulate HPV18-LCR activity [8-15]. AP1 represents a key regulatory protein in the maintenance of E6/E7 gene expression in almost all HPV types hitherto investigated [14,16]. HPV18-LCR contains two identical AP1 binding sites (TGACTAA) in opposite orientations, one located in the promoter (nucleotides 7792–7798) and the other one in the enhancer (nucleotides 7607–7613) [17]. Both sites are essential for HPV18 transcription from the early P_105 _promoter [8,13,16,18] and AP1 transactivation is required for tumour promotion *in vivo *[19]. AP1 also appears to be involved in negative regulation of HPV transcription, since treatment of HPV16 immortalized human keratinocytes with the anti-oxidant pyrrolidine-dithio-carbamate (PDTC) selectively reduced the amount of viral mRNA by blocking transcription initiation, an effect that is profoundly associated with alterations of the AP1 heterodimerization pattern [20]. Therefore, there is considerable interest in identifying compounds able to down-regulate AP1 activity for the treatment of HPV related malignant lesions.

Glycosaminoglycans (GAGs) are unbranched polysaccharide chains composed of repeated disaccharide sequences that consist of sulphate groups in various positions [21]; these groups give the GAG chains a net negative charge. In 1989, Regelson reported that polyanionic substances such as heparin, a member of the GAG group, are tumour inhibitors [22]. This effect may result from the binding of anionic heparins to a wide range of proteins and molecules, thus affecting their biological activities. As a consequence, heparins have a wide variety of biological properties other than their anticoagulant effects, and those properties may interfere with the malignant processes [23]. Heparin can affect proliferation, migration, and invasiveness of cancer cells in various cell types, including those derived from epithelial cells [24-27]. It has been shown that heparins selectively inhibit the phosphorylation of mitogen activated protein kinases [28,29], and there is direct evidence that heparin penetrates into the cell nucleus and causes inhibition of Fos-Jun/AP1 activity as a direct result of its nuclear localization in HeLa cells [30]. Additionally, heparin and the heparinoids dextran sulfate and pentosan polysulfate, potently and selectively inhibit the *in vitro *replication of herpes simplex virus 2 (HSV-2), cytomegalovirus (CMV), AIDS virus (HIV), vesicular stomatitis viruses, respiratory syncytial, influenza type A, Sendai, Junin, and Tacaribe viruses [31-35]. The growth of rat vascular smooth muscle cells transformed by SV40 was also inhibited by heparin [36].

In this work, we use the heparin analogue GAG-hed to determine its effect on HPV18 early expression in two murine models and in cultured HeLa cells. Our results demonstrate that GAG-hed inhibits tumoral growth in a model generated using HeLa cells in *nu/nu *mice. A direct inhibitory effect of GAG-hed on the activity of HPV18-LCR was also noticed, as shown by the inhibition of β-galactosidase expression in HPV18-LCR-LacZ transgenic mice. Additionally, in HeLa cells, Northern and RT-PCR analysis showed that GAG-hed treatment resulted in a suppressive effect on E6/E7 viral expression and also GAG-hed has a significant negative effect on cell viability in HeLa cultures. Finally, this product also inhibited the sequence specific binding of the nuclear factor AP1 to HPV18-LCR. When tested by an *in vitro *approach, we found a blockade in protein/DNA binding activity due to GAG-hed treatment. All of these data suggest a potential antitumoral and antiviral application for GAG-hed.

## Methods

### Materials

GAG-hed was isolated from porcine intestine mucosa and obtained from PROBIOMED laboratories (México). In comparison with standard heparins, this molecule has a higher average in the amount of sulfate groups with a molecular weight ranging from 3–14 kDa.

### Cell culture and cell proliferation assay

HeLa cells (HPV18 positive cervical carcinoma derived cell line), C33-A (HPV negative cervical carcinoma derived cell line) and SW480 (colorectal adenocarcinoma) were routinely cultured in Dulbecco's modified Eagle's medium (DMEM, Invitrogen Gaithersburg, MD) supplemented with 10% fetal calf serum, with the appropriate antibiotic mix at 37°C in a 5% CO_2 _atmosphere. Culture media was replaced every two days.

Cell viability was measured by the MTT (3-(4, 5-dimethylthiazolyl-2)-2, 5-diphenyltetrazolium bromide) reduction assay, performed as first described by Mosmann [37]. Cells were seeded on a 24-well plate in 500 μl culture media with or without GAG-hed, incubated for indicated times and analyzed (quadruplicates). After incubation of the cells with the MTT reagent (5 mg/ml) for approximately 4 h, an isopropanol: HCl solution was added to lyse the cells and solubilize the coloured crystals. The samples were read using an ELISA plate reader (wavelength of 630 nm) Opsys MR (Dynex Technologies).

### Athymic mice

Athymic nude mice were obtained from Instituto Nacional de la Nutrición animal facility, bred and maintained in the animal facilities at the Instituto Nacional de Cancerología of México. Athymic female mice, 5–6 week old were housed (3 animals/cage, n = 12) in holding rooms that were kept at 21–25°C, and 40–60% relative humidity.

### Tumorigenicity

HeLa cell cultures at 80% of confluence were trypsinized, washed twice and resuspended in 200–500 μl phosphate buffered saline (PBS). HeLa cells were subcutaneously (S.C.) inoculated in the dorsal position in only one inoculation place in each female nu/nu mice. After a period of 10 to 16 days, when small nodules were already palpable, GAG-hed was administrated at three different concentrations of GAG-hed, two doses per week, all applied intra-tumoral and tumour growth was monitored. Tumours were measured twice a week and tumour volume in cubic millimetres was calculated as vol = (d_1_d_2_d_3_π)/6, where the width of the tumour is used twice as d_1 _and d_2 _and the length as d_3 _[38]. The two dimensions were measured at least twice using Venire callipers.

### Transgenic mice and GAG-hed treatment

Female mice expressing the LacZ gene under the control of the HPV18-LCR were reported previously [39,40]. Transgenic mice (line 406) were crossed back to C57Bl/6J X C3HeB/FeJ F1 no transgenic strain and hemizygous 3 to 6 months old F1 females were obtained for our experiments. A volume of 50 μl of GAG-hed (10 mg/ml) or physiological solution as placebo, were introduced into the vagina of the female mice with a small probe attached to an insulin syringe. Doses were applied at noon and at night along 6 days. The seventh day vaginal smears were spread in slides for fixation and staining with hematoxylin-eosin and the phase of the oestrous cycle was determined by microscopic examination of the cells. Immediately after, the animals were sacrificed, dissected, and organs were frozen for transgene activity quantification. Two groups of three females each in estrogenic phase (proestrous-oestrous) were selected for these experiments.

### β-galactosidase assay

Determination of β-galactosidase activity was described by Cid-Arregui et al. [41]. Briefly, crude extracts from organs were prepared homogenizing with polytron in PM-2 buffer containing 33 mM NaH_2_PO_4_, 66 mM Na_2_HPO_4_, 0.1 mM MnCl_2_, 2 mM MgSO_4_, 40 mM β-mercaptoethanol, pH 7.3 and centrifuging twice for 10 min in a microfuge, discarding the pellet and lipid layer. Reactions were carried out with 200 μg of protein and 800 μg of ONPG (o-nitrophenyl-β-D-galactopyranoside) as substrate in a final volume of 1 ml of PM-2, and incubated at 37°C for 1 h. Color development was measured at A_420 _against a blank without protein. β-galactosidase-specific activities were calculated, after subtracting the initial absorbance of the reaction, using the formula: units = 380 X A_420_/time (min), such that 1 unit is equivalent to the conversion of 1 nM of ONPG per min at 37°C [42]. For whole-organ staining vaginas were dissected and immediately fixed in 1% formaldehyde, 0.2% glutaraldehyde in PBS (pH 7.3) for 1 h at room temperature and then washed 4–5 times in PBS. Staining was performed with 1 mg/ml of X-Gal (5-bromo-4-chloro-3-indolyl-β-D-galactopyranoside; Sigma) in 0.01% sodium deoxycolate, 0.02% Nonidet P-40 in PBS at 30°C for 6 h in the dark. Finally, a second fixation was performed in 1% formaldehyde/2% glutaraldehyde in PBS.

### Northern blot

Total RNA was extracted from HeLa cells employing the Chomczynski method [43], with minimal modifications. RNA was run in de-naturalizing gels, checked for equally loading RNA amounts, transferred to Hybond-N nylon membranes and fixed by baking membranes to 80°C for 2 h. As probe we used the fragment BamH1/EcoR1 containing the viral early region, obtained from plasmid pBR2.4 reported by Lazo [44]. Labelling was performed by nick translation (Amersham Biosciences, Buckinghamshire, UK) using ^32^P-dCTP. Filters were hybridized for 16–24 h at 65°C, washed and analyzed by autoradiography.

### RT-PCR

Total RNA was isolated from confluent C33-A cells, and from treated and non treated HeLa cells. Treatment was performed with increasing GAG-hed concentrations for 48 h. Total RNA was extracted from cells using the Trizol method (Invitrogen Gaithersburg, MD). All samples were treated with RNAse-free DNAse (Invitrogen Gaithersburg, MD) to prevent genomic DNA contamination during PCR amplification. First strand cDNA was prepared from 1 μg of total cellular RNA using First Strand cDNA Synthesis Kits with oligo dT as a primer (Invitrogen Gaithersburg, MD). All PCR reactions were carried out in a 25 μl total volume containing 1 X PCR buffer, 4 mM MgCl_2_, 200 μM dNTP, 100 ng each of forward and reverse primers (Sigma Aldrich St. Louis, MO), and 1 unit of Taq polymerase in a Peltier Thermaln Cycle (Perkin-Elmer). Amplification conditions for both E6/E7 and β2-microglobulin were as follow: after an initial 94°C incubation for 3 min, reactions were amplified for 35 cycles at 94°C for 30 s, 58°C for 60 s and 72°C for 90 s. The reactions were then incubated at 72°C for 10 min. PCR amplification products were separated on a 1% agarose gel and visualized by ethidium bromide staining. Primers used to amplify the E6/E7 gene were as follows: forward, 5'-TGTCAAAAACCGTTGTGTCC-3', and reverse, 5'-GAGCTGTCGCTTAATTGCTC-3' [45]. Primers used to amplify the β2-microglobulin were: forward 5'-ACCCCCACTGAAAAAGATGAGTAT-3', and reverse, 5'- ATGATGCTGCTTACATGTCTCGAT-3'.

### Cell cycle analysis

For determination of cell percentage in each phase of the cell cycle a standard procedure based on the established method of whole-cell staining with propidium iodide (PI) was followed [46]. Briefly, cells were trypsinized, fixed and permeabilized in 70% ice cold ethanol to make them accessible PI. Once fixed, cells were rinsed with PBS and stained with a PBS solution containing 0.1% Triton X-100, 0.2 mg/ml DNase free RNase A and 0.02 mg/ml of PI. Triton X-100 was included to decrease the cell loss resulting from electrostatic cell attachment to tubes and the RNase to digest the double stranded sections of RNA that might stain with PI. Measurements were done on a FACSCalibur Instrument (Becton-Dickinson) with an excitation of 488 nm (argon-ion laser line). Data were analyzed using the ModFit LT V2.0 (PMac) DNA content histogram de-convolution software.

### Apoptosis determination

Translocation of phosphatidylserine (PS) to the external surface of the membrane was determined using the Annexin V-FLUOS staining kit (Roche Applied Science) according to the manufacturer's instructions. HeLa cells were treated for 12 hours with staurosporine (1 μM, Sigma) as a positive control of apoptosis induction. The percentage of early apoptotic and apoptotic lysed cells was determined on a Becton-Dickinson FACS Vantage SE flow cytometer.

Chromatin condensation and/or nucleus fragmentation were investigated morphologically by DAPI (4',6-Diamidine-2'-phenylindole dihydrochloride) staining. Apoptotic cells were estimated by counting cells on UV microscopy after staining.

### Electrophoretic Mobility Shift Assay

We performed gel mobility shift experiments (EMSA) utilizing nuclear extracts of HeLa cells prepared as previously described [47]. All buffers contained a protease inhibitory cocktail to prevent nuclear factor proteolysis. Protein concentration was measured by the Bradford method using the Bio-Rad protein assay reagent [48]. Double stranded oligonucleotides were end-labelled with [α-^32^P] dATP or [α-^32^P] dCTP (3000 Ci/mMol) and Klenow enzyme. Labelled oligonucleotides were incubated with up to 7–8 μg nuclear protein in a reaction mixture with 2x BDG buffer containing 24 mM HEPES, pH 7.8, 20% glycerol, 0.1 mM EDTA, 8 mM MgCl_2_, 20 mM KCl, 2 mM dithiothreitol, 4 mM spermidine for 10 min on ice; 0.5 μg poly [dI-dC] was added as unspecific competitor (Pharmacia Biotech). After probe addition, the reaction mixtures were incubated for 10 min on ice, electrophoresed in 6% polyacrylamide gels using a low ionic strength 0.5X TBE buffer. The gels were dried and exposed to an autoradiography film. The double stranded oligonucleotides used were:

AP1 from HPV18 [49] 5'- CTAGAATATGACTAAGCT-3' CTTATACTGATTCGAGATC;

SP1 (*bona fide *SV40) 5'-CTAGATTCGATCGGGGCGGGGCGA-3' TAAGCTAGCCCCGCCCCGCTGATC;

Egr-1 (*bona fide*) [50] 5'-CTAGGATCCAGCGGGGGCGAGCGGGGGCGA-3' CCTAGGTCGCCCCCGCTCGCCCCCGCTGATC

## Results

### GAG-hed inhibits tumour growth in nu/nu mice

**Figure 1:**
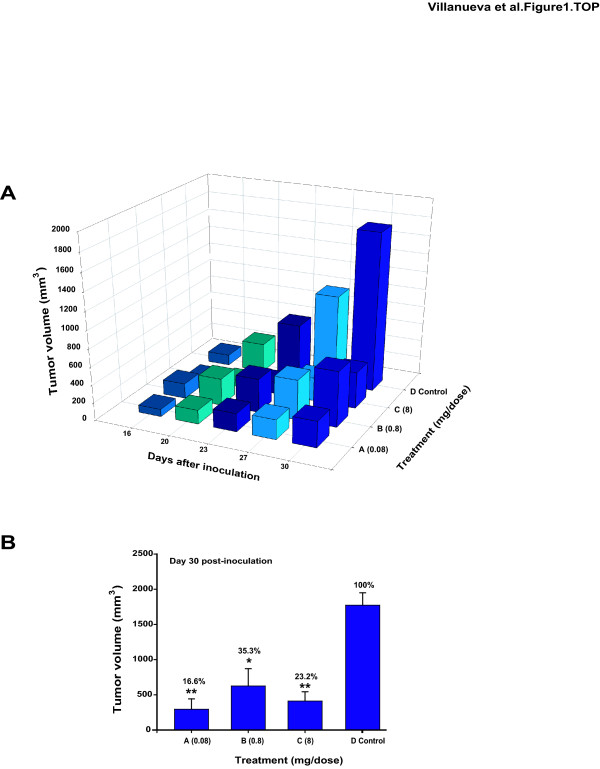
**GAG-hed affects growth rates in a HeLa tumoral model induced in athymic female mice**. Nude female mice were subcutaneously inoculated on the dorsal part with HeLa cells (12 mice total, three mice per group); each animal received only one cell injection (1 × 10^6 ^cells in suspension, in a total volume of 20 μl). On established tumours, GAG-hed doses applied were: 0.08 mg/dose, group A; 0.8 mg/dose, group B; and 8 mg/dose, group C. Control animals were injected with saline solution and are denoted as group D. Panel A; Data showing the average tumour volume determined from day 16 after cell inoculation (day taken as starting point for treatment) to day 30th. Tumour volume was determined by two dimensions measurement: width and length. Z axis: average of total tumour volume (expressed in mm^3^) of all animals (n = 3); Y axis: days after HeLa cells inoculation; X axis: applied GAG-hed treatment. (B) Comparison and statistic analysis values at day 30th under differential dose application. *p < 0.05, **p < 0.005, Student's *t*-test in comparison to saline treated control.

In order to induce tumours derived from HeLa cells, 1 × 10^6 ^cells were injected into athymic female nude mice. Tumours developed within 16 days following subcutaneous cell injection. Treatment began at day 16, when tumours in all mice in the groups A, B and C received intra-tumoral GAG-hed, 0.08, 0.8 and 8 mg per injected dose, respectively. Control group D received only intra-tumoral saline injections; three mice were included in each group. Tumour size was measured twice a week and tumour volumes calculated as stated in Methods. Mice that received saline injections developed tumours whose size was measured as 100% of tumour growth. Much less accelerated growth was observed over time for mice that were treated with GAG-hed, a comparison of the tumour volume is plotted in Figure 1A. At day 30, the mean tumour volume +/- SE was calculated for each group of independent experiments and homogeneous data were evaluated by an unpaired Student's *t*-test in order to detect overall differences between control and treated groups on the final day. Statistically significant differences in tumour size were noticed in animals receiving treatment, irrespective of applied doses. Tumour growth was maintained below 37% in treated animals compared with non treated controls (Figure 1B). These results suggest that GAG-hed inhibits tumour growth rates but growth inhibition did not show a dose-dependent behaviour.

### In vivo effect of GAG-hed on HPV18-LCR activity

**Figure 2:**
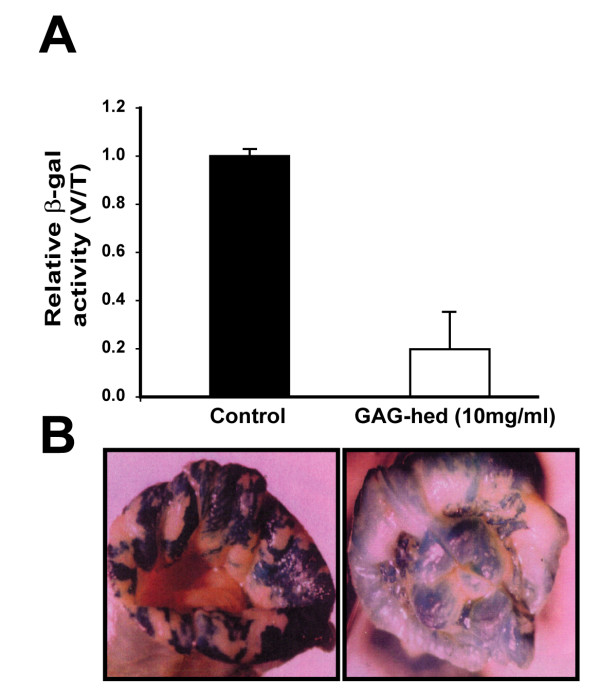
**GAG-hed down-regulates the HPV18 LCR activity in transgenic mice**. Female transgenic mice were used to test GAG-hed effects on the expression of lacZ reporter gene whose transcription is driven by HPV18-LCR. GAG-hed vaginal washes (10 mg/ml, 50μl) were applied twice a day for 6 days. Six vaginal and tongue specimens were stained or tested for β-galactosidase activity. (A) Relative activity is expressed as the relationship between β-galactosidase units in vaginal (V) and tongue (T) tissues (3 mice) for the unique concentration employed compared with the effects of saline application (3 mice). (B) Images obtained from vaginal samples stained for β-galactosidase using X-Gal. Left panel, vaginal sample treated with saline solution (control); right panel, vagina treated with GAG-hed.

The mechanisms of how GAG-hed may inhibit the development of a large tumour mass are not well understood. We wondered if part of this effect may be mediated by a direct inhibition of HPV18-LCR transcriptional activity. To further investigate if the GAG-hed effect seen in HeLa induced tumours is affecting papillomavirus oncogene expression, we used transgenic mice carrying one integrated copy of the HPV18-LCR-lacZ reporter gene per genome. This model has proven to be useful in determining the effect of different exogenously administrated compounds on HPV expression [39,40]. The encoded β-galactosidase enzyme was detected in epithelia of specific organs (e.g. vagina, cervix) when tissue extracts were tested in proper assays containing substrates such as ONPG or X-Gal. Under the same experimental conditions, no β-galactosidase activity was detected for normal mice tissues (vagina, cervix, tongue)[40]. As seen in Figure 2A, the activity of the HPV18-LCR in the genital tract of female mice dropped to one-fifth after local treatment with GAG-hed as compared to mice treated only with saline solution. Here, we decided to report the obtained values as the ratio of values recorded from vagina divided by the endogenous activity found in the tongue of the same mice (e.g. vagina/tongue ratio) to reflect a more realistic result. As expected, transgene expression was specifically inhibited in vagina and cervix after heparin treatment as shown by an intense blue indigo colour which turns into a lighter one in the organ pieces shown in Figure 2B. The activity values of saline-treated mice correlate well with those previously found in untreated females (data not shown), and is quite different when compared with non-transgenic mice tissue, where activities are virtually zero [40].

### Effect of GAG-hed on E6/E7 expression in HeLa cells

**Figure 3:**
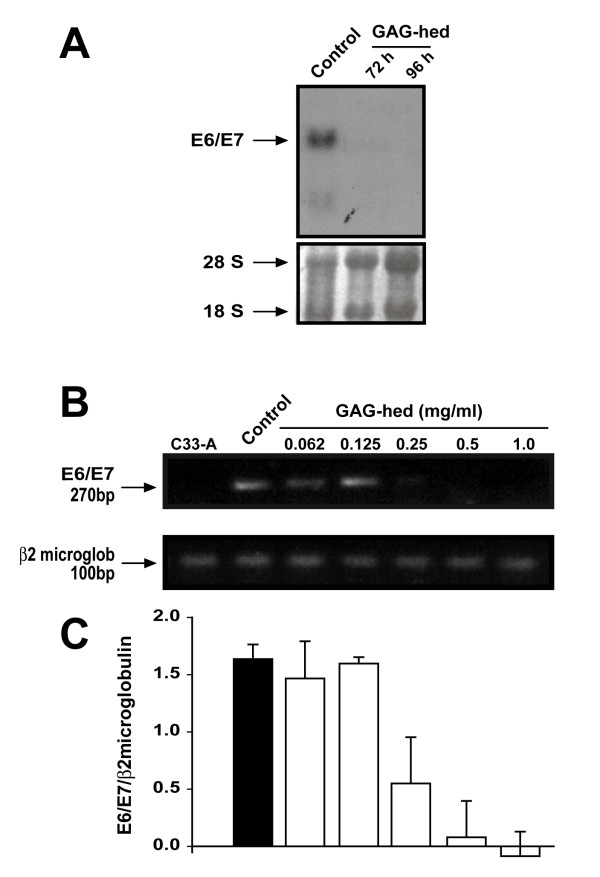
**GAG-hed causes repression of HPV18 E6/E7 transcription in HeLa cells**. (A) Northern blot was performed with total RNA obtained from HeLa cells treated with 2 mg/ml of GAG-hed and harvested 72 or 96 h later. During treatment, cells were grown in media with 2% serum. Control cells were harvested after 72 h. Gel image showing similar RNA loading in gel was taken before transfer and shown as reference. (B) Cells were treated with the indicated increasing concentrations of GAG-hed and total RNA was extracted after 48 h. RT-PCR assays were performed and DNA products were separated on agarose gels. RNA used as negative control was from C33-A cell line. E6/E7 transcript is denoted as a 270 bp band (top); same mRNAs were used to test the expression of β2-microglobulin, denoted as a 100 bp band (bottom). (C) Plot represents densitometryc data normalized against microglobulin optical density.

With the demonstration that GAG-hed inhibits LCR-directed expression *in vivo*, we focus our attention on the possibility that GAG-hed treatment may inhibit HPV18 E6/E7 viral transcription. In order to verify this, E6/E7 mRNA expression was detected by Northern blot and RT-PCR in GAG-hed treated and control HeLa cells. Total RNA was extracted from cells after variable times and treatment doses. For Northern blot, we treated cells with a fixed concentration of GAG-hed (2 mg/ml) and harvested them after 72 or 96 h. As a probe, we used a labelled DNA fragment taken from plasmid pBR2.4 which contains the HPV18 early viral region [44]. In Figure 3A, the E6/E7 transcript is undetectable after GAG-hed treatment. A gel image showing similar RNA loading was taken before transferring (Figure 3A, lower panel). For RT-PCR, HeLa cells treated with five increasing concentrations of GAG-hed were employed. The HPV-negative cervical carcinoma cell line C33-A was used as a negative control. Figure 3B shows a dramatic decrease in the levels of HPV18 E6/E7 mRNA after GAG-hed treatment. This decrease is evident for cells treated with GAG-hed at concentrations as low as 0.25 mg/ml, while repression is complete at higher concentrations (0.5 and 1.0 mg/ml; Figure 3B, top). As can be seen in the lower panel, the expression of β2-microglobulin was not affected by treatment. Plotted data were obtained by scanning and quantifying of labelled bands. Optical densities for E6/E7 bands were normalized to the β2-microglobulin signal (Figure 3C).

### GAG-hed affects cell proliferation rates but did not induce apoptosis

**Figure 4:**
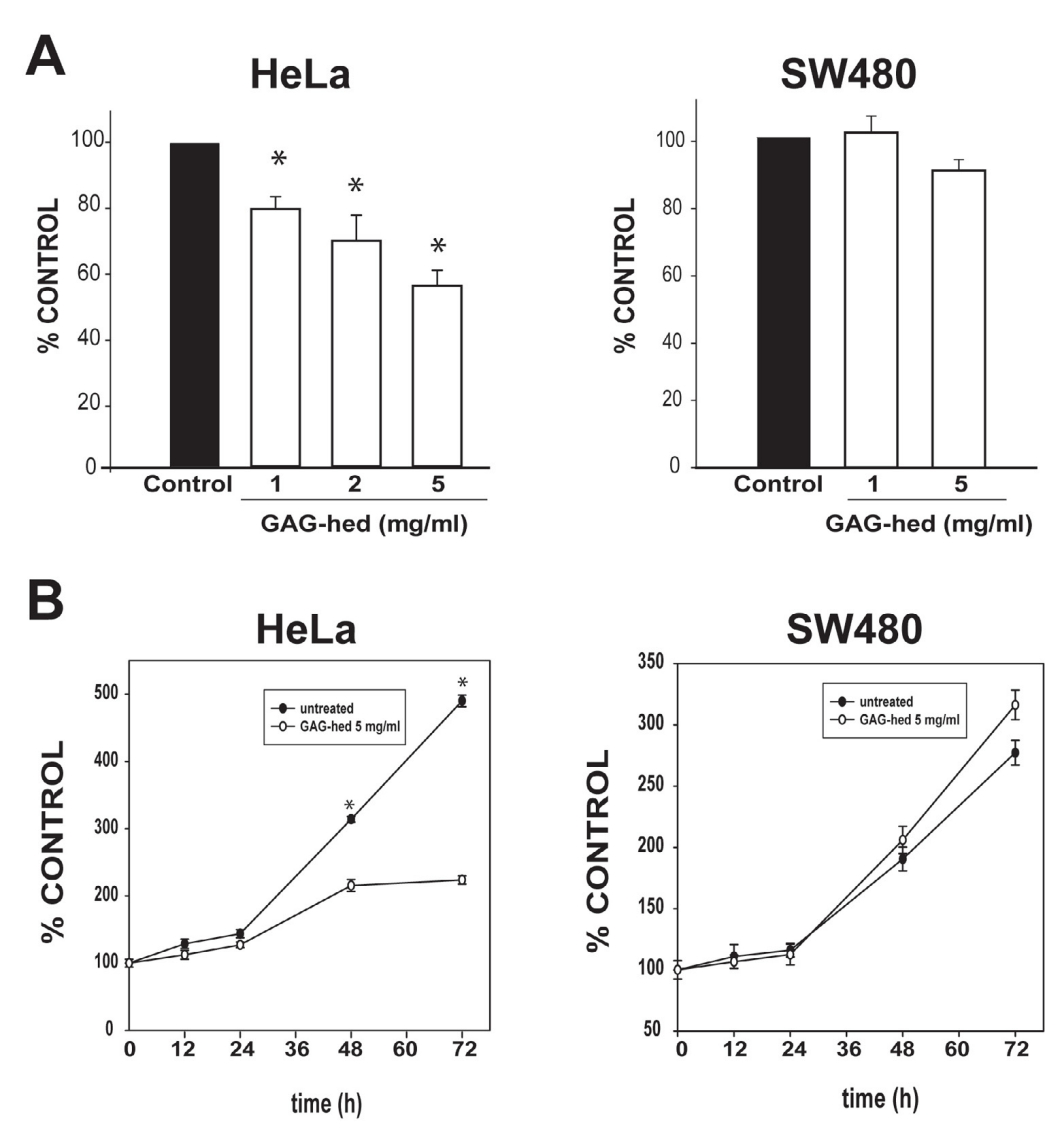
**Cell viability and proliferation rates were affected by GAG-hed**. HeLa and SW480 cell viability was assessed by the MTT method. Cells were seeded on a 24-well plate. In (A), cells were treated with increasing doses of GAG-hed for 48 h. In (B), after GAG-hed treatment (5 mg/ml) cells were incubated and analyzed at 12 h intervals for 3 days. *, differences were significant when compared with equivalent untreated cells (control) in all cases (Student's *t*-test p < 0.001; n = 4).

In concordance with other reports [51], the same suppressive effects of GAG-hed over E6/E7 expression shown by Northern blot and RT-PCR analyses can be translated into a significant decrease in proliferation rates for HeLa cell cultures, when this parameter was assessed by the MTT method (Figure 4A). As a comparison, the SW480 colorectal adenocarcinoma cell line was tested using the same experimental conditions. No significant changes in cell proliferation were noticed in this case, supporting a correlation between loss of E6/E7 expression and GAG-hed effects. Additionally, cell proliferation assays demonstrated that HeLa cells treated with GAG-hed proliferated at a lower rate than those untreated. Again, SW480 cells did not show any differences in their proliferative rates when those treated with GAG-hed were compared to untreated (Figure 4B).

**Figure 5:**
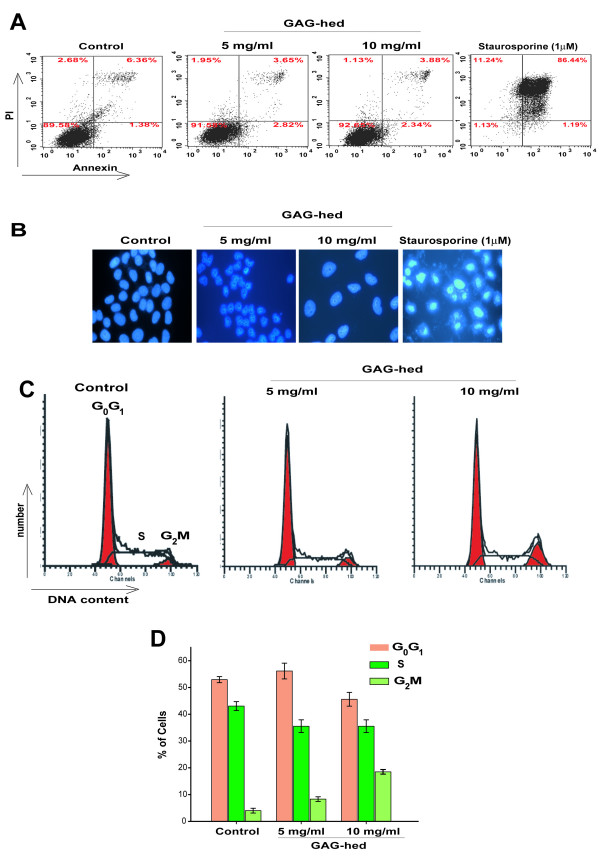
**GAG-hed arrest cell cycle but did not induce apoptosis**. (A) 1 × 10^6 ^cells were treated with GAG-hed (5 and 10 mg/ml) for 48 h. The cells were then permeablized, stained for Annexin V, and stored on ice until analyzed by FACS. The upper panels show representative examples of control HeLa cells either untreated (left) or treated with staurosporine to induce apoptosis (right). Increased Annexin V staining was seen in HeLa cells only in the presence of staurosporine. In all panels, cells in the lower left quadrant are alive, cells in the lower right quadrant are in early apoptosis, in the upper right are in late apoptosis, and cells in the upper left quadrant are dead. Percentage of total signal within the quadrant is indicated. (B) Fluorescent microscopic analysis of cells stained with DAPI. Forty-eight hours after GAG-hed treatment the cells were fixed, stained with DAPI and analyzed for morphological characteristics associated with apoptosis. (C) Cell cycle distribution in HeLa cells treated with GAG-hed. Histograms are derived from a single experiment that was repeated three times with similar results, that in (D) are expressed as percentages for each of the three cell cycle phases, mean ± SE of three independent experiments.

At this point, it was important to determine if GAG-hed treatment induced apoptosis. Apoptotic cells were identified by transport of phosphatidylserine to the membrane surface (Figure 5A) and by microscopic observation of DNA fragmentation in the nucleus (Figure 5B). We noticed the absence of apoptosis, by using Annexin-V with flow cytometry and DAPI nuclear staining. As a positive control, we treated HeLa cells with staurosporine, recording a clear change in the flow-cytometry output, indicative of apoptosis induction (Figure 5A). Characteristic condensed nuclei of apoptotic cells were clearly visible when cells were treated with staurosporine and stained with DAPI (Figure 5B).

Furthermore, flow-cytometric analysis showed that cells treated for 48 h with GAG-hed underwent a change in cell cycle progression. Cells accumulated in the G_2_/M phase after GAG-hed treatment depending on applied dose (Figure 5C and 5D). The percentage of cells in G_2_/M increased from 4 % in the control to 18.43% in those treated with 10 mg/ml of GAG-hed.

### Sequence specific binding of nuclear factor AP1 is inhibited by GAG-hed

**Figure 6:**
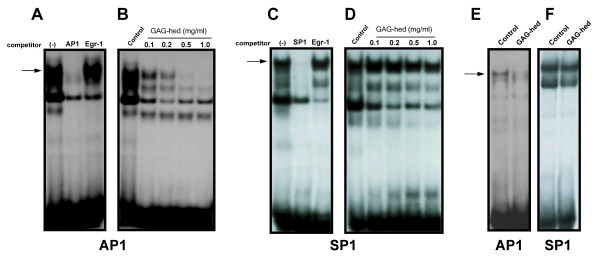
**GAG-hed blocks AP1 binding**. AP1 DNA binding was performed using electrophoretic mobility shift assays (EMSA) with oligonucleotides containing the HPV18 AP1 binding sequence and the SV40 SP1 *bona fide *site. Oligomers were end labelled with [α-^32^P] dATP or [α-^32^P] dCTP. Nuclear extracts obtained from HeLa cells were exposed to GAG-hed at indicated concentrations for 10 min before labelled probe addition (Panels B and D). On panel A and C, complex specificity is denoted by competition assays with a 100 fold excess of indicated oligonucleotide competitor. In panel E and F, AP1 and SP1 binding respectively, was tested with the same nuclear protein extract obtained from HeLa cells treated *in vivo *with 5 mg/ml of GAG-hed and harvested 48 h post-treatment. Control indicates untreated cells.

**Figure 7:**
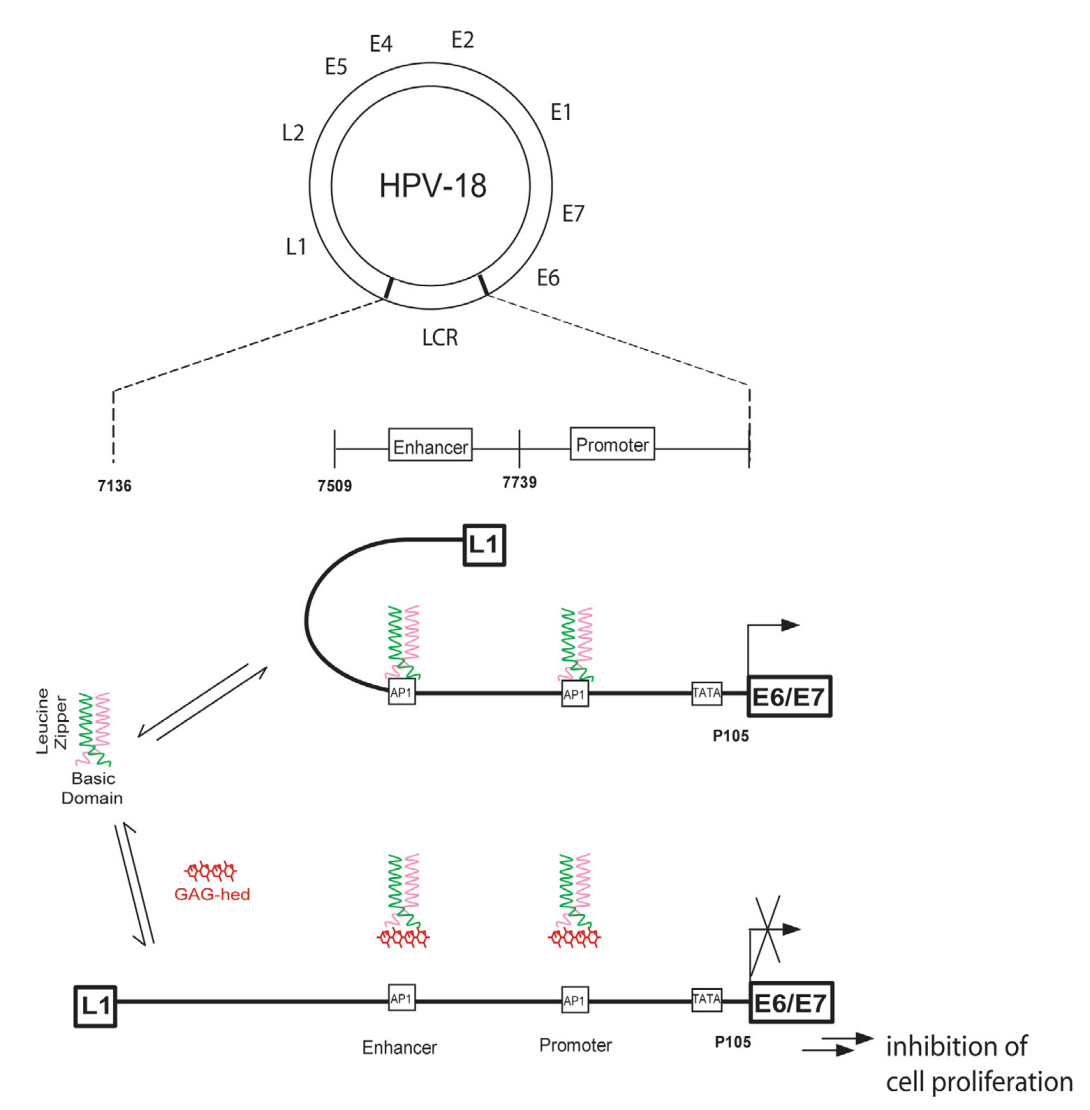
**Proposed model for GAG-hed modulation of AP1 transcription factor in HPV18 LCR**. Fos-Jun/AP1 transcription factor complex is bound off in the presence of GAG-hed. GAG-hed-AP1 complex is rendered stable and unable of binding to DNA. Model in based in data reported in previous work [30,69] and particularly adapted to HPV18-LCR. Circular HPV18 genome showing the early and late open reading frames (ORF). The LCR has been expanded to show clearly the promoter and enhancer regions in front of E6 and E7 genes. The AP1 binding sites located in the promoter and the enhancer are indicated. Apart from the TATA sequence, the other known binding sites for transcription factors in the LCR of HPV18 have not been indicated.

Among heparin effects, a direct inhibition of AP1 binding has been reported [30]. To further investigate the molecular mechanism implicated in the inhibition of viral oncogene transcription shown in our study, we tested the possibility that GAG-hed blocks the binding of AP1 transcription factor to HPV18-AP1-binding sequences using an electrophoretic mobility gel shift assay (EMSA). We used HeLa nuclear extracts for EMSA analysis and selected as a probe an oligonucleotide containing the sequence of the AP1 elements from HPV18-LCR. The two AP1 binding sites in HPV18 are located at nucleotides 7795 and 7613, at positions -171 and -349 with respect to the P_105 _starting site. Both sites contain identical sequences 5'-TGACTAA-3' that have been shown to be recognized by JunB/c-Fos heterodimers in HaCat cells [14]. The binding specificity for the AP1 and SP1 complexes was determined in competition experiments by the addition of a 100-fold molar excess of either unlabeled homologous or heterologous oligonucleotides, before adding end-labelled DNA probe. Competition experiments are displayed in Figure 6A and 6C for AP1 and SP1, respectively, where homologous unlabeled oligonucleotide effectively competes for the major binding factors present in HeLa nuclear extracts. In contrast, under similar conditions, a heterologous oligonucleotide such as the *bona fide *site for Egr-1 (Early growth response gene transcription factor 1) did not compete for binding in both cases. In Figure 6B, nuclear extracts from HeLa cells shifted ^32^P-labeled AP1 oligomers from HPV18, and in the presence of increasing amounts of GAG-hed, specific complexes are progressively lost. To exclude unspecific effects of GAG-hed, nuclear transcription factor SP1 was tested using the very same nuclear extracts in similar EMSA studies. During employment of SP1 labelled oligonucleotide, no inhibitory effect on complexes was observed, indicating that GAG-hed does not interfere with the DNA binding of SP1 (Figure 6D). Finally, in Figure 6E, we show that GAG-hed treatment caused the very same effects on AP1 when treatment was applied to cells in culture, before harvesting them for nuclear extract processing. In this case, labelled AP1 oligomer was not retained when cells were treated with GAG-hed. Therefore, by blocking AP1 binding, GAG-hed may inhibit HPV18 expression and E6/E7 associated uncontrolled cell proliferation (see Figure 7). Note the lack of effect in Figure 6F when SP1 was tested.

## Discussion

The present study provides a closer look into the effects of heparinoids over the expression of HPV type 18, employing different methods and experimental systems. All assays were performed with GAG-hed, a highly sulphated heparinoid formulation which is heterogeneous in molecular weight (3–14 kDa), that has been used here to provide four lines of evidence suggesting that this compound blocks AP1 binding to HPV18 LCR. First, HeLa derived tumours developed in athymic mice grew significantly less under treatment with GAG-hed. Second, in transgenic female mice containing the HPV18-LCR-LacZ transgene, GAG-hed negatively affects the LCR activity based on the reduction of β-galactosidase reporter expression after treatment. Third, GAG-hed abolished the expression of HPV18 E6/E7 genes in HeLa cells and cell proliferation was profoundly affected. Finally, GAG-hed also reduced the binding of AP1 to its DNA sequence in HPV18 LCR, separately shown both by direct *in vitro *application and when cells in culture were previously treated for two days.

In HPV containing carcinoma cells, the GAG-hed antiproliferative effect may also be the result of an antiviral effect. The transcription of the HPV18 P_105 _promoter, which controls expression of the E6 and E7 transforming genes, is regulated by a combination of viral and cellular factors. The transcription factor AP1 is essential for the activity of this promoter, a finding mainly based on the observation that mutation of the corresponding binding sites within the HPV18-LCR completely abolishes P_105 _promoter activity in human keratinocytes [14,16]. Indeed, it has been shown that AP1 also plays a central role in positive transcriptional regulation of several other human pathogenic HPVs [52,53]. JunB is an important factor in HPV18 transcription in keratinocytes. In nuclear extracts prepared from human keratinocytes, JunB was the predominant Jun component bound to the DNA probe containing the same cis element we tested here (Figure 6) [14]. It is therefore reasonable to assume that all selection mechanisms during HPV-linked carcinogenesis which enhance AP1 activity are required for HPV to exert its function as a DNA tumour virus. In HPV-positive carcinoma cells, GAG-hed causes a strong inhibition of AP1 binding to specific sites located on the viral LCR (Figure 6), which probably explains the decrease in E6/E7 mRNA synthesis. Diminution in β-galactosidase activity observed here (Figure 2), in addition to Northern blot and RT-PCR experiments in HeLa cells (Figure 3), are both highly suggestive of a direct affectation in AP1 regulatory activity within HPV18 LCR. Diminished expression in E6/E7 transcripts has been shown to be associated with inhibition of proliferation of cervical tumour cells [7,51,54]. Consistently, we noticed a significant loss in HeLa proliferation rates (Figure 4), which may be associated with the importance of AP1's role in E6/E7 expression. AP1 also plays a crucial role in cell proliferation [55], regulating gene expression in the pre neoplastic-to-neoplastic progression in cell culture models [56-63]. It is also possible that AP1 binding is blocked at the level of several cellular promoter regions of genes involved in cell proliferation, enhancing the antitumoral capabilities of GAG-hed. Heparin may have multiple targets for its antiproliferative activity; it can bind to Fos and Jun peptides, rendering the AP1 factor unable to bind DNA. Heparin selectively blocks the induction of immediate-early genes like *c-fos *and *c-jun *and other genes which are involved in cell cycle progression, including *c-myc*, *c-myb*, tissue plasminogen activator and ornithine decarboxylase [28]. Heparin suppresses PMA induced expression of *c-jun *and Jun B mRNA and protein in baboon VSMC [28]. Blocking *c-fos *and *c-jun *transcription by heparin is particularly important for transit through the cell cycle. However, the most significant effect of heparin on Jun family members occurs post-translationally. As Jun B is synthesized, it is converted to a higher molecular weight form by phosphorylation(s). Heparin prevents the transition to the higher molecular weight species, which is presumably the active form of Jun B, suggesting that heparin- mediated inhibition of Jun B may account for the reduced AP1-binding to the phorbol ester response element found on promoter regions upstream of the collagenase and tissue-type plasminogen activator (TPA) genes [64]. Highly specific effects of heparin on signal transduction pathways involving MAPK, PKC and CaMK II have also been reported and may be reflected in the effects of heparin on gene regulation.

GAG-hed treatment affects cell proliferation but did not induce apoptosis (Figure 5). In HPV positive cells, p53 levels are regulated by the continuous expression of E6. The E6 oncoprotein has been shown to recruit the cellular ubiquitin-protein ligase E6-AP to target the tumor-suppressor protein p53 for ubiquitin-proteasome-mediated degradation [65,66]. Treatment with antiE6 resulted in down-regulation of E6/E7 mRNA and an increase in p53 levels accompanied with a significant decrease in the growth rate [51]. Part of the mechanism by which p53 blocks cells at the G_2 _checkpoint involves inhibition of Cdc2, the cyclin-dependent kinase required to enter mitosis. Cdc2 is inhibited simultaneously by three transcriptional targets of p53, Gadd45, p21, and 14-3-3σ [67]. We observed an increase in the percentage of HeLa cells in G_2_/M phase together with a slight decrease in the number of cells in G1 and S phases with GAG-hed treatment, which may be associated with a recovery in cell cycle control by the increase in p53 after lowering E6/E7 expression. Current work in our laboratory is exploring this point.

GAGs are acidic and highly negatively charged molecules, which interact with a large number of proteins and basic molecules through ionic and hydrogen bonding interactions [68]. There is direct evidence for heparin incorporation into cell cytoplasm and its presence in the nucleus [30,69]. There is also evidence that heparan sulfate and glycosaminoglycans in the Extracellular Matrix and on the cell surface can be internalized by cells while bound to receptors. The uptake of heparin involves complexation and internalization with fibroblast growth factor and fibroblast growth factor receptor [70,71]. Once in nuclei, heparin binds AP1; in 1992, Busch showed that ^125^I-labeled-heparin also binds directly to Fos and Jun peptides. In line with these observations, important changes in the levels of "free" transcription factors can be observed as a result of heparin internalization. It has been recently shown that heparin complexation with transcription factors may result in their inability to bind DNA regulatory elements, increasing factor levels in the cytoplasm and nuclei and culminates in apoptosis and cell death [69,72]. Heparin also inhibits other important transcription factors in VSMC, such as *c-myb *and Oct-1, which presumably play a role in cell proliferation [73-75].

## Conclusion

Model depicted in Figure 7 summarizes our current understanding of how GAG-hed may block HPV18. Based on previous studies [30,69,72], plus our present data, we suggest a possible molecular mechanism by which GAG-hed displays the effects described here, supporting the idea of heparinoids as plausible anti-viral and anti-tumoral drugs.

## Competing interests

The author(s) declare that they have no competing interests.

## Authors' contributions

RV carried out the molecular studies and drafted the manuscript. NMP and ACA carried out the transgenic mice assays. ICS and EGV participated in mRNA evaluation. RT performed cell cycle analysis and apoptosis assays. ELB, AGC and PG participated in study design and coordination and helped to draft the manuscript. All authors read and approved the final manuscript.

## Pre-publication history

The pre-publication history for this paper can be accessed here:
